# The physician's Alzheimer's disease management guide: Early detection and diagnosis of cognitive impairment, Alzheimer's disease and related dementia

**DOI:** 10.3934/publichealth.2022047

**Published:** 2022-09-27

**Authors:** Allison B. Reiss, Donna de Levante Raphael, Nathaniel A. Chin, Vivek Sinha

**Affiliations:** 1 NYU Long Island School of Medicine, Mineola, NY 11501 USA; 2 National Memory Screening Program, Alzheimer's Foundation of America, 322 Eighth Avenue, New York, NY 10001, USA; 3 Wisconsin Alzheimer's Disease Research Center (ADRC), Madison, WI 53726; 4 Belleview Medical Partners, Alexandria, VA 22314 USA

**Keywords:** Mild Cognitive Impairment, Alzheimer's disease, dementia, brain health, primary care physicians, health care clinicians, Alzheimer's knowledge, early detection, diagnosis and management, cultural competency, barriers to diagnosis, reversible dementia, irreversible dementias

## Abstract

Primary care professionals play a critical role in the care of their patients. In clinical practice, early detection and diagnosis of Mild Cognitive Impairment, Alzheimer's disease and related dementia are often missed or delayed. Disclosure of diagnosis is not timely or not revealed. Though the methods that could improve early detection and diagnosis have remained the same over the decades with little change, they provide opportunities for early intervention, treatment and improvement in patient care. Emerging research suggests that though the disease process begins years prior to the clinical diagnosis, the healthcare system and health care professionals remain distant and reluctant to provide the service of annual cognitive assessment, which has been recommended by the Medicare program for older adults aged 65 years and older. Findings support that Alzheimer's disease and related cognitive impairments have gone under detected, underdiagnosed and undertreated. This article seeks to provide valuable and equitable information in the form of a clinician's guide for removing the barriers to early detection and diagnosis of cognitive impairments and offers an unprecedented opportunity to improve the clinical outcomes and care of older adults with various levels of cognitive decline, including mild cognitive impairment, Alzheimer's disease, and related dementias. This article provides information on understanding and addressing the challenges faced by health care professionals, including primary care clinicians; removing the barriers to cognitive assessments; educating this professional group on the importance of brain health, early detection, and diagnosis for their older adult patients; and providing these professionals with the ability to transfer their knowledge into more defined care planning. Until cognitive screening has been fully accepted and implemented for the optimal the care of older adults, health-related efforts should include the promotion and education of brain health, early detection, and diagnosis in the education of health care providers.

## 1. Introduction

Primary care providers (PCPs), healthcare systems and practices vary regarding their practices' relevant information. This toolkit aims to provide options to PCPs with approaches and tools they can select to remove the barriers to conducting cognitive assessments for their patients. This toolkit will have a section for each step necessary to initiate and increase cognitive awareness, detection and diagnosis of cognitive impairment and medical care.

It is recommended by the Centers for Medicare & Medicaid Services that each individual 65 years and over receive an annual evaluation of one's cognitive health status. Medicare's Annual Wellness Visit (AWV) for those aged 65 years and older requires direct cognitive assessments as one of the several components for preventative care [1]. This quality metric is vital because it can improve the health outcomes of individuals and their families. If the identification of cognitive problems can be diagnosed earlier, a thorough investigation into reversible causes can begin. Additionally, patients can be educated on lifestyle behaviors that may help improve or maintain cognitive function. Additionally, we can gain valuable insight into cognitive and behavioral issues as the individual experiences them within their healthcare system. Cognitive screenings will allow physicians and other clinicians to work with patients and families to proactively develop care plans and improve the quality of life for all. Cognitive assessments should be part of every senior's annual wellness visit.

Initial cognitive screenings typically take a few minutes and may include questioning the patient and the patient's family, observing the patient's interactions or using short verbal or written tests [2]. These initial memory screenings do not give a diagnosis. A diagnosis requires a full cognitive assessment. Due to various challenges and barriers in screenings, physicians may often skip this evaluation. Therefore, many older adults are usually diagnosed only when severely impaired (i.e., with a dramatic change in daily activity). Due to this delay, it is often too late for the individual to plan for their future, seek effective treatments that are only effective in the early stages of the disease or enter a clinical trial. Individuals have the right to understand what is happening to them and make informed decisions based on their current situation. Having a diagnosis and knowing of a disease affecting one's memory can help the individual and their family effectively engage with their health care team and their familiar environment.

## 2. Materials and methods

We systematically searched eight databases to secure relevant information pertaining to topics related to aging, gerontology, and geriatrics: ProQuest, Google scholar, PubMed, CINAHL, PsycINFO, PsycARTICLES, MedLINE and the National Institute on Aging. To remain relevant, we sought articles published over the past 5 years with minimal papers prior to the 5 years. The search strategy applied incorporated 40 selected articles using the Boolean method. This method allowed the use of a combination of modifiers with keywords such as MCI, Alzheimer's disease, dementia, brain health, assessments, primary care physicians. We extracted relevant literature for this research article that resulted in the following keywords: Mild Cognitive Impairment, Alzheimer's disease, dementia, brain health, primary care physicians, health care clinicians, Alzheimer's knowledge, early detection, diagnosis and management, cultural competency, barriers to diagnosis, reversible dementia, and irreversible dementias. The Patient/Population, Intervention, Comparison and Outcomes (PICO) question and framework were useful in guiding the search and locating relevant keywords for the article.

## 3. Results

**Figure 1.**
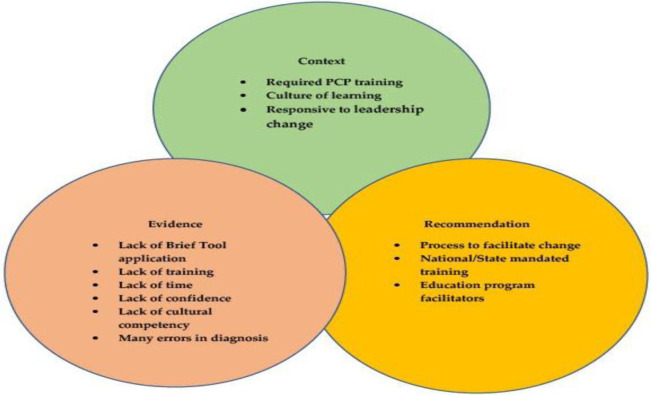
Main challenges encountered in clinical practices that overlap.

Results revealed that there were extensive papers on best practices for clinicians, practice guidelines for early detection and the importance of early diagnosis and caring for one's patient with cognitive decline. Figure 1 (Main Challenges Encountered in Clinical Practices That Overlap) shows the main overarching issues within the medical field. Context, evidence, and recommendations listed in the table below affect the early detection and diagnosis and care management of the patients. Through interpretation, it was agreed that, for optimal outcomes for the patient and their family caregivers, the clinician required the knowledge and understanding of the disease and what care management and support should look like for their patient with dementia. The steps to follow would be to initiate a conversation with patients, early detection, and diagnosis, followed by a multidisciplinary evaluation to rule out dementia. If the result is dementia, then that would be followed by providing a collaborative care plan with recommendations from other support teams followed by monitoring and disease management.

### 3.1. Cognitive impairment

#### 3.1.1. Dementia

Dementia is the loss of cognitive functioning. Dementia is not a disease but a group of symptoms caused by other conditions [3]. Dementia is the most prevalent of brain disorders in older adults [3]. Globally, dementia is one of the leading causes of disabilities in older adults [3]. Dementia is the umbrella term used to describe the group of symptoms causing memory loss, language loss, reduced judgment, reduced problem-solving skills and other thinking skills severe enough to interfere with activities of daily living and social and occupational functioning.

#### 3.1.2. Subjective cognitive decline (SCD)

SCD is referred to as self-reported information of increased changes in cognition such as memory loss and confusion. SCD is a type of cognitive impairment. SCD is one of the earliest recognitions of developing symptoms of cognitive decline, Alzheimer's disease and related dementia [4].

#### 3.1.3. Mild cognitive impairment (MCI) vs. Dementia

MCI can develop for multiple reasons. MCI is not considered dementia [5]. However, people with MCI may develop dementia. Mild Cognitive Impairment can be an early sign of Alzheimer's. Not everyone with MCI will develop the disease. A person with dementia can experience more critical cognitive challenges than a person with MCI. The differences between MCI and dementia are related to the severity of the individual's symptoms.

#### 3.1.4. Subjective cognitive decline (SCD) vs. Mild cognitive impairment (MCI)

Subjective cognitive decline (SCD) is self-perceived by a person who is cognitively normal in the absence of objectively measured cognitive deficits [6]. Mild cognitive impairment (MCI) is viewed as a transitional stage that rests between the cognitive decline in normal aging and dementia [7].

#### 3.1.5. Alzheimer's disease (AD)

Alzheimer's disease is the most common form of dementia. According to the National Institute of Aging (NIA), Alzheimer's is currently the seventh-leading cause of death in the United States [6]. It is a progressive brain disorder that slowly destroys memory, thinking skills and eventually the ability to carry out the simplest tasks, such as one's activities of daily living (ADLs). Age is the leading risk factor for Alzheimer's disease [8].

#### 3.1.6. Dementia vs. Alzheimer's disease

Dementia is the general term applied to the group of symptoms negatively impacting memory [8]. Dementia is not restricted only to memory and encompasses a decline in cognitive function as well as alterations in mood, behavior and personality [9]. At the same time, Alzheimer's disease is a specific and progressive disease causing slow but permanent memory loss while affecting cognitive function.

#### 3.1.7. Signs and symptoms of Alzheimer's disease

Although each individual is unique, experts have identified common warning signs of Alzheimer's disease, including

- Memory loss, especially of recent events, names, places and other new information
- Confusion about time and place
- Struggling to complete familiar and complex tasks such as managing medications, managing finances, driving or household chores
- Trouble finding appropriate words, for example, in a sentence
- Difficulties in judging situations
- Changes in mood and personality

### 3.2. Stages of Alzheimer's disease

The most popular of AD stages set by the National Institutes of Health's National Institute on Aging describes the disease in three stages: early (mild), middle (moderate) and late (severe).

#### 3.2.1. Early (Mild)

In this stage, people may

- Forget words or misplace objects
- Forget something they just read
- Ask the same question over and over
- Have increasing trouble making plans or organizing
- Not remember names when meeting new people

#### 3.2.2. Middle (Moderate)

In this stage, people may have

- Increased memory loss and confusion
- Problems recognizing family and friends
- Continuous repeating of stories and questions, favorite wants (e.g., foods, places, songs etc.), or motions
- Decreased ability to perform complex tasks (e.g., planning dinner) or handle personal finances (e.g., paying bills)
- Delusions – such as thinking they need to go to work, even though they no longer have a job
- Lack of concern for hygiene and appearance
- Requirements of assistance in choosing proper clothing to wear for day, season or occasion

#### 3.2.3. Late (Severe)

In this stage, there is almost total memory loss.

- The individual may recognize faces but forget names.
- The individual may mistake a person for someone else.
- There is a strong need for holding something close for tactile stimulation, nurturing, companionship and comfort.
- Basic abilities such as eating, walking and sitting up fade during this period; the individual may no longer recognize when they are thirsty or hungry and will need help with all basic activities of daily living.

### 3.3. Risk factors of dementia

A person's physical and lifestyle behaviors often factor in raising their chances for dementia, including:

- Age
- Dementia in the family history
- Depression
- Brain injury
- Strokes
- Lack of exercise
- Diabetes
- Obesity
- Poor diet, which refers to the failure to provide the body with adequate amounts of and forms of nutrients for ultimate health
- Cardiovascular disease
- Sleep apnea
- Smoking and heavy alcohol use
- Infection of the brain (such as syphilis and meningitis)

### 3.4. Common causes of dementia

The most common causes of dementia are as follows.

#### 3.4.1. Degenerative neurological diseases

Alzheimer's disease

- Dementia with Lewy Bodies
- Huntington's disease
- Parkinson's disease
- Some forms of multiple sclerosis

#### 3.4.2. Vascular disorders

- Stroke, aneurysmal SAH or cerebral amyloid angiopathy

#### 3.4.3. Other common causes of dementia include

- HIV/AIDS-associated neurocognitive disorders (HAND)
- Chronic alcoholism
- Tumors
- Vitamin B12 deficiency
- Carbon monoxide poisoning
- Cerebral anoxia
- Hypothyroidism
- Subdural hematoma
- Hypothyroidism, hypoglycemia

### 3.5. Cognitive screening and diagnosis

In the past, a definitive Alzheimer's disease diagnosis could not be made until after death if a brain autopsy is completed. However, in the past decade, there have been new diagnostic approaches such as the amyloid PET, CSF (cerebrospinal fluid) biomarkers and tau and plasma tests, which have been used in the clinical diagnosis of Alzheimer's disease. These tests can be used to accurately diagnose early-stage Alzheimer's disease. Some of these tests correlated well with pathological processes and can make accurate diagnosis of Alzheimer's disease prior to autopsy. Blood biomarkers, however, are less predictive [9],[10]. Today, physicians can also diagnose Alzheimer's with reasonable certainty by conducting tests that can eliminate other causes of memory loss and confusion and by reviewing how the patient's symptoms align with the disease [9],[10]. A cognitive screening requires several reliable tests to screen for Alzheimer's or related dementia. A screening test should not be used as a substitute for a complete diagnostic evaluation [9],[10]. There is no single diagnostic test than can determine an Alzheimer's diagnosis [9],[10]. A complete clinical diagnostic evaluation often includes the screening of other physicians who use various approaches and tools to help give an accurate diagnosis, such as:

- Geriatrician
- Geriatric psychiatrist
- Neurologist
- Neuropsychologist

#### 3.5.1. A complete diagnosis or clinical evaluation involves

- Disclosure of symptoms consistent with the symptoms of Alzheimer's disease or related dementia
- Diagnosis of other health conditions or other symptoms to rule out reversible conditions
- Review of medical records and medications (to include prescriptions or over the counter drugs, vitamins and supplements)
- Family history
- Mental Status Exam (Used to evaluate cognitive functioning objectively)
- Laboratory Tests (Such as Blood work or a urinalysis). Used to screen for infections and other medical conditions that could hinder a person's thinking ability.
- Imaging (Such as a CT, MRI or PET scan)
- Differential Diagnoses (An evaluation for reversible conditions that may mimic Alzheimer's disease (depression, delirium)

These tests can also help to differentiate between the various types of dementia, such as

- Parkinson's disease
- Frontotemporal dementia (Pick's disease)
- Vascular dementia

#### 3.5.2. What is an assessment?

It is the collection of symptoms from both the person and the informant. The collection of symptoms includes cognitive signs and functional symptoms, with screening for objective cognitive change through a brief test to determine if there is a potential presence of impairment [11],[12]. This will also include a “full workup”/complete medical examination to include mood screening, more focused cognitive history, medication review, laboratory test (looking for reversible causes) and brain imaging [11]–[13]. Some of this is likely done by a specialist.

### 3.6. Challenges and barriers to cognitive screening

Though there are structured processes for other health conditions, there is none for cognitive diseases. There are no specific standardized tests or guidelines established for cognitive assessments [14],[15]. The cognitive assessment and diagnosis approach has been reactive when patients raise issues or self-report their cognitive experiences or challenges [14],[15]. This approach can make cognitive screenings challenging in a clinical setting. Some of these challenges may include the following:

- The clinician does not have special training for cognitive screenings
- Screenings cause a delay in the clinic's workflow
- Time limitations, fear of giving a diagnosis
- Lack of cultural competency in their patient's understanding of the cognitive changes
- Not knowing how to start the conversation
- Alzheimer's disease stigma
- Fear of harm to the older patient by conducting a cognitive assessment that may lead to depression or anxiety
- Discrimination

With these challenges and barriers, many health care providers remain hesitant to initiate the concerns of cognitive testing with their older patients. Providers instead are waiting for the older adult or family member to initiate the conversation about their problems. Physicians are in an ideal position to observe potential signs of cognitive decline and ask pertinent questions. As the provider to the individual, you may have a long-established relationship with the individual and their family [16],[17]. When the patient is concerned about any changes in their memory functioning, they would most likely take the concern to their providers [16],[17]. However, older adults will rarely initiate the conversation about their cognitive challenges for various reasons [16],[17]. Some reasons may include:

- Fear
- Cultural perceptions of the disease
- Stigma
- Historical experiences
- Not believing there is a benefit to knowing about the disease
- “Why bother? Dementia is incurable.”

### 3.7. How to start the conversation

It is essential not to ignore the changes in an older adult's memory, mood, behavior or personality. It is critical to take the concerns seriously and assess the patient as early as possible. You are more likely to be able to determine the potential cause for the consideration of brain functioning changes. When memory problems are reported by the patient or observed by a family member or yourself, these changes should be documented in the patient's chart, followed by a screening and assessment [18],[19].

For the best care outcome, it is essential to know and explain to the patient and their caregiver that not all cognitive changes or problems result from Alzheimer's disease and that various other possible causes affect the brain's optimal functioning [18],[19]. Some of these causes may be side effects from medications or a combination of drugs, thyroid conditions, untreated depression or anxiety, metabolic and endocrine changes, obstructive sleep apnea, strokes, traumatic brain injuries, infections like urinary tract infections (UTIs), pneumonia, vitamin deficiencies, delirium caused by other illnesses, stress or, finally, a brain-specific disease like Alzheimer's disease. Many of these conditions that cause memory impairment are treatable or reversible. Other causes of cognitive impairment cannot be reversed; however, the symptoms may be treated, and quality of life can still be optimized.

Alzheimer's disease specifically and dementia in general have become terms that terrify many patients, hampering their willingness to disclose information about their symptoms to their physicians. That makes even more important how physicians approach a cognitive screening test conversation. Physicians must approach the topic of cognitive screening by maintaining the focus on the importance of getting a baseline test, looking for reversible causes and recommending lifestyle changes that can lead to improved overall function and wellbeing. Treat cognitive screenings as a necessary test like you would for blood work or cancer screening. This approach provides the patient with the understanding that a baseline test should be updated at least yearly. Evidence for routine annual cognitive screenings has been insufficient [18],[19]. The initiation of a cognitive assessment relies mainly on self-reported symptoms, so physicians must engage patients and their families in healthy, proactive conversations about cognition as a normalized topic during their annual wellness visits.

### 3.8. Importance of cognitive assessments of older adults

Americans aged 65 and older are expected to double from 52 million in 2018 to 95 million by 2060. This older adult population has become more racially and ethnically diverse [19],[20]. It is essential to know that the population has changed, with more older Americans than before. Because Alzheimer's disease is an age-related disease, many people develop Alzheimer's or already have it. The issues with cognitive impairment in older adults can result from various possible causes, as seen in the table below.

**Table 1.** Common causes of cognitive impairment, 2022.

Common cause	Description	Notes/Examples
Medication side-effects	Many medications interfere with proper brain function.	Such as Sedatives, Tranquilizers, and Anticholinergic medications.
Hormonal problems	Such as Thyroid hormones	Imbalances in estrogen or other sex hormones.
Vitamin deficiencies	Brain function is affected by low levels of vitamin B12, other B vitamins and folate.	-
Delirium	State of worse than normal mental function.	Common in hospitalized older adults due to infection and other health problems.
Infections	Not as common in older adults as the other causes, but certain chronic or acute infections can affect brain cells directly.	If the infection is outside of the brain (pneumonia or UTI), it is considered delirium).
Vascular injury	“Vascular” damage to neurons means damage caused by problems with the blood vessels.	Such as strokes or some form of cerebral small vessel disease.
Traumatic injury		Head injuries are also associated with temporary or longer-lasting cognitive impairment.
Neurodegenerative condition	Neurodegenerative conditions tend to damage and kill neurons slowly.	This can cause mild cognitive impairment and then eventually dementia. The more common neurodegenerative disorders include Alzheimer's disease, Lewy-Body disease, Parkinson's disease and frontotemporal degeneration. Though not a neurodegenerative condition, patients with small vessel disease may experience MCI then a dementia status.
Substance abuse	Both acute intoxication and chronic overuse of certain substances can impair brain function.	Such as alcohol, illicit drugs or even prescription drugs.
Psychiatric illness	Most psychiatric conditions can cause problems with memory, thinking and concentration.	Paranoia, depression, anxiety, mental illness, bipolar disorder, schizophrenia.
Metabolic imbalances	Abnormalities in one's blood chemistry leading to diabetes, heart disease, stroke, high blood pressure or dementia.	Abnormal levels of blood sodium, calcium or glucose; kidney or liver dysfunction.
Toxins	Toxins are another potential cause of cognitive impairment.	Research is ongoing about the effects of toxins people may be exposed to, such as pesticides, contaminants in drinking water, air pollutants, heavy metals and others.

**Table 2.** Neurodegenerative conditions – Progressive dementias.

Condition	Warning signs
Alzheimer's disease	Increased memory loss, confusion, inability to learn new information, difficulty with language, reading and writing and decline in judgment.
Parkinson's disease	Tremors/shaking of limb, hand or fingers. Rigid muscles, impaired posture and balance, loss of automatic movements and speech and writing changes.
Lewy body dementia	Hallucinations or delusions, changes in movement, sleep problems, behavior shifts.
Frontotemporal disease	Apathy, unwillingness to talk, personality/mood changes, depression, lack of inhibition, lack of social tact, obsessive/repetitive behaviors.
Vascular dementia	Confusion, difficulty concentrating, slowed thinking, a decline in the ability to analyze a situation and reduced ability to organize thoughts or actions.
Creutzfeldt-Jakob disease	Personal changes, memory loss, blurred vision or blindness, insomnia, difficulty speaking and swallowing.
Mixed dementia	Slowness of thought, difficulty planning, difficulty understanding and concentrating, changes in mood and behavior, memory and language loss.

It is essential to understand that there are reversible and non-reversible conditions and to first rule out the curable/treatable conditions before diagnosing Alzheimer's disease or related dementia. As shown in Table 1, there are many causes of cognitive impairment. Some of these cognitive impairment conditions, such as medication side effects and depression, can be reversed or improved with treatment options. Table 2 defines Neurodegenerative Conditions, also known as progressive dementias. Such conditions as Alzheimer's disease cannot be reversed. Symptoms, however, can be treated over a period of time, usually in the early stages of the disease. It is essential to acknowledge that cognitive assessments can offer the individual and their family opportunities to prepare for future predictable changes and address safety concerns that come along with the progression of the disease [20].

Many individuals with memory loss or behavioral changes choose to get a diagnosis to understand the challenges they are experiencing and what to expect in the progression of the disease. In contrast, at times, others and their families are very reluctant to reveal their concerns because of the fear of the diagnosis of Alzheimer's disease, the challenges of stigma upon diagnosis and the future it foreshadows [21]. This reluctance often occurs with individuals from different socio-cultural backgrounds, and the physician or other clinician needs to understand their beliefs and traditions to treat them further [21]. By engaging these individuals and their families and asking why or why not, you can then understand their position on the disease and their reluctance to undergo diagnosis and treatment and eventually move closer to the diagnosis. However, it is important to have a conversation to engage the patient and understand the differences. In this conversation, the physician or provider can explain the benefits of getting the assessment to discover what may be causing the individual's health concerns.

Pharmacological treatment options for memory loss related to Alzheimer's disease and other cognitive symptoms only help to control the symptoms, and these treatments are limited. None of these treatments can reverse, slow or cure the course of the disease. The pharmacological management approaches listed here are those currently FDA (Food and Drug Administration) approved for cognitive and functional deficits, including behavioral symptoms. The most common treatments for dementia are cholinesterase inhibitors: Donepezil (Aricept®), Rivastigmine (Exelon®) and Galantamine (Razadyne®) can help with symptoms, particularly in the earlier stages, and can lead to delayed institutionalization and reduced caregiver burden [22]. Medications for moderate and severe Alzheimer's disease are Glutamate regulators such as Memantine (Namenda®) and Cholinesterase inhibitor + glutamate (Namzaric®), which can help with symptoms, particularly in the more severe stages [22]. A study from a Harvard memory clinic showed that patients showed a slower downward decline in cognitive symptoms and functional changes after three years based on questionnaires [22],[23]. This example suggests that while these medications don't treat underlying disease pathology, they can have a meaningful subjective impact on people as they are changing.

The newest pharmacological approach that was recently approved in 2021 was Aducanumab. This amyloid beta-directed antibody mediation is also known as Aduhelm. This medication was designed to decrease the amount of beta-amyloid and remove specific forms of beta-amyloid that accumulated into plaques in the brain. Aduhelm was designed to be administered to patients intravenously (IV) with infusions every four weeks. Aduhelm had several adverse effects on patients, such as bleeding in and on the brain's surface, various allergic reactions, and temporary swelling of the brain, including symptoms of nausea, confusion, dizziness, vision changes and headaches. This drug came with a lot of controversy. The main points of controversy were that there was insufficient evidence of efficacy, that the medication created false hope and that the high price would negatively impact the patients financially.

Another option for providing care is the use of non-drug approaches. Some individuals and their families are adamant about not resorting to pharmacological treatments. This decision must be respected. There are many options for non-drug approaches that can manage behavioral symptoms. It is highly recommended to promote the use of non-drug approaches to your client first before issuing a prescribed medication.

### 3.9. Reversible dementias

Reversible dementias are conditions associated with cognitive or behavioral symptoms that can be resolved with treatment once the primary cause is treated. As seen below in Table 3 (Reversible dementias), dementia-like symptoms can be reversed with treatment. Many potential causes of reversible dementias have been identified, resulting in reversible neurocognitive function impairment in older adults. There are, however, treatable dementias that may not be curable due to the delay in diagnosis.

#### 3.9.1. Common causes of reversible dementia

**Table 3.** Reversible dementias.

Reversible dementia	Causes	Symptoms
Normal pressure Hydrocephalus	Referred to as “water on the brain.” – a condition where extra spinal fluid gets trapped in the brain.	Confusion & memory loss, urinary incontinence, balance and walking problems.
Nutritional Deficiencies/Vitamin B12 deficiency	Poor diets, other health problems such as pernicious anemia or Crohn's disease and older age cause reduced ability to absorb this vitamin.	Similar to Alzheimer's disease – memory loss, behavior changes, agitation.
Thiamine/ Wernicke/Korsakoff syndrome	Thiamine deficiency (Vitamin B1). It is often due to alcohol abuse.	Ataxia, muscle weakness, diminished/hyperactive reflexes, urinary or fecal incontinence, loss of vision.
Thyroid disorders	Hypothyroidism and hyperthyroidism.	Memory loss, difficulty finding the right word, concentrating, poor spatial organization and slower visual processing.
Sleep deficits	Chronic sleep deprivation causes memory and overall cognition to decline.	Significant impact on the ability to think or reason.
Brain lesions	Risk factors: radiation exposure, age, obesity, pesticide exposure and genetic factors.	Memory loss, poor judgment, personality changes, impulse control, headaches, seizures and hearing. Vision, speech and physical changes.
Delirium	Urinary Tract infection or pneumonia.	Increase in challenging behaviors, more lethargy, increased confusion
Subdural hematomas	It can develop from what is a minor bump on the head.	Confusion, lethargy, difficulty with speech and headaches
Depression/Mood changes	-	Symptoms are similar to early signs of dementia, memory loss and problems with word recall.
Medication reactions	Some medications cause cognitive issues: Cholesterol-lowering statins, Chemotherapy rugs, Pain medications, Anti-Anxiety medications and Sleeping pills.	
Alcohol/Tobacco/Drug use	Cause of type of anemia.	Iron Deficiency
Dehydration	A person's awareness of thirst declines with age.	
Anoxia/Hypoxia	A state of oxygen deprivation. Caused by a heart attack, monoxide poisoning or severe asthma.	
Withdrawal from drugs	Discontinuing use of some prescriptions.	Confusion and disorientation.

### 3.10. Why early detection matters

Over the years, research has been gradually revealing that early detection of cognitive change can help to extend the longevity of the patient and enhance the quality of life for those who are eventually diagnosed with a cognitive disease and their family caregivers [24]. An Alzheimer's disease diagnosis often doesn't occur until the symptoms are advanced, and a person is in the later stages of dementia. It would be better if the diagnosis occurred much earlier, during the mild cognitive impairment (MCI) stage. Undiagnosed, misdiagnosed and unmanaged Alzheimer's disease can result in unnecessary suffering for the individual with the disease and their family. Moreover, the cost of care can be much more expensive than adequately managed Alzheimer's disease. Another benefit of an early diagnosis is that it gives the person with Alzheimer's disease the opportunity to participate in clinical trials and making their own life decisions. Medication can assist with specific symptoms, but clinical trials can match patients to studies focused on their condition, allowing them to participate in research that could alter the disease.

Though there is no cure for brain diseases like Alzheimer's, some medications and treatment plans can assist in making a big difference in daily functioning and disease development. We understand that we can preserve our health, including cognitive health, through positive lifestyle changes. Proactive lifestyles matter, and the earlier they are put into effect, the better. It is never too late to start. Even a person who has been currently diagnosed with Alzheimer's can maintain their functioning longer under a program of proactive interventions.

A cognitive screening test is a tool and not a guaranteed solution for a complex problem. However, screening tools serve a crucial role in documenting early signs of cognitive impairment. The tools may allow physicians to discover patterns in brain function decline before they've progressed into dementia. Often, these tools are meant to identify dementia itself so that people can understand what's happening to them and then make the best decision they can with that information. The next step is tracking down the cause of the cognitive decline, playing an active role in care coordination and investigating if medication and lifestyle changes may slow the disease progression. By engaging patients in their overall health conversations and focusing on how their overall health supports brain functioning, you can turn a scary discussion and possible realization into an opportunity for patient empowerment and better disease management.

Physicians play an essential role in identifying individuals at risk. Identifying and recommending various lifestyle changes for an adult in midlife may prevent or slow down the disease, and a timely diagnosis may be helpful and start a meaningful conversation with the patient [25]. Early diagnosis and intervention is an optimal strategy for patient care because the individual's level of functioning can be preserved for a longer period of time.

### 3.11. Benefits of early Alzheimer's disease diagnosis to the patient

- Early diagnosis explains the signs, symptoms, and behavioral changes that the patients are experiencing. This diagnosis provides an answer for their suspicions.
- An early diagnosis offers access to the right services and supports to help individuals take control of their condition and experience, live independently in their own home for longer and maintain a good quality of life for themselves, their family, and caregivers.
- A good quality of life in the early stages of the disease can be maintained for several years.
- Diagnosed individuals have the opportunity to plan ahead while they have the capacity to do so.
- Individuals also have the opportunity to participate in their own legal, financial and future support/care options and treatment and make their wishes known to family members and the medical team.
- It is only through receiving a diagnosis that patients can access treatments and support to improve their cognition and enhance their quality of life.
- Early diagnosis offers caregivers the time to adjust to the changes in function, personality, mood and behaviors that come with the stages of the disease and their transition to the caregiver role, especially those who are spouses or adult children, where the roles change tremendously.
- Caregivers who are prepared tend to adapt better, feel more competent to provide care, experience fewer psychological problems such as anxiety and depression and delay the institutionalization of their loved ones with Alzheimer's disease.

### 3.12. Benefits of early screening for the physician

Should the cognitive screening result be negative, concerns may be alleviated until later. Screening should be conducted annually to monitor changes from the baseline established from the initial assessment. If the screening result is positive, then further medical evaluation is required: The next step would be to identify the cause of the individual's cognitive changes (such as depression, delirium, urinary tract infection, thyroid imbalance, medication side effects, metabolic and endocrine imbalance, anxiety or Alzheimer's or another dementia) [26]. This may result in:

- Treatment of the underlying disease or health conditions
- Managing comorbidities effectively
- Referring the person and their caregiver to a specialist in behavioral health. A specialist may be able to provide the individual with memory tools used to become more organized to manage their changing symptoms better
- Addressing safety issues
- Engaging the person to create or update their advance directives and long-term care
- Ensuring the person has support through a care network, support and services
- Assistance with medical, legal and financial matters
- Ensuring the person and their caregiver receive the appropriate information, referrals, and other support for coping with Alzheimer's disease
- Working with the individual and their care partner on developing care strategies to improve their quality of life, making necessary modifications to the person's lifestyle and home safety and managing emotional experiences related to the Alzheimer's diagnosis
- Encouraging both the individual and caregiver to participate in clinical research
- Ensuring that the caregiver receives the compassion and appropriate information, referrals, and support in coping with the Alzheimer's disease diagnosis and stress management

### 3.13. Cognitive screening test options

There are several widely known cognitive screening tests available. So, which cognitive screening tests are recommended for use in the Medicare Annual Wellness Visit? What circumstances require further testing? The Medicare Annual Wellness Visit does not recommend a preferred cognitive test. Whenever possible, it is best to use standardized, validated tools because they are required for some elements of the assessments. Not all tools have been formally tested for validity and uptake in actual primary care physician practices. Ideally, cognitive assessment tools should be:

- **Practical.** Minimum time and effort to complete in the primary care setting. Ideally administered by a non-physician such as a medical assistant or nurse.
- **Retrievable.** Searchable at point of care and included in any electronic health records.
- **Scorable.** Results are illustrated in single numbers.

Specific screening tests are often based on the physician's observations and the patient's responses. A cognitive screening test can be administered in less than 10 minutes and may reveal crucial data indicating the need for further testing. Patients who willingly confirm awareness of their symptoms or who have scores that indicate a possible memory problem on the initial screening test should be given a more detailed assessment and be referred for a complete cognitive evaluation. Patients who deny symptoms observed by clinicians or reported by family members or cannot confirm their symptoms should still be given a brief structured assessment, such as the **MMSE, Mini-Cog, MIS, MOCA** or **GPCOG**.

Importantly, collecting symptoms, which can be accomplished through tools such as the **Short IQCODE** or **AD8**, allows you to gather more insight, especially if they are administered concurrently. Collecting symptoms can help with monitoring responses to therapeutic interventions. The goal of collecting and assessing symptoms would be to gather data that is valid, reliable, responsive to change, discriminating and unbiased. Important considerations would be the method of data collection, which should be accessing the symptoms, the timing of the assessment and the methods used to assess multiple symptoms.

Patients may view the topic of cognitive decline as frightening, especially when they're speaking about a disease that has no actual treatment and no cure. On the other hand, physicians may see the topic of cognitive decline as a medical health discussion that seeks to track the change in the condition. When your patients' cognitive screening test scores justify further exploration, taking a proactive viewpoint in the exam room is essential. This can make all the difference to the patient. Physicians need to explain to their patients that a cognitive screening test is an assessment, not a diagnosis. While screening tests may suggest a problem with brain function, further testing will be required to confirm a problem exists and determine what is causing it.

As a physician, it is essential to engage patients in the process of learning about and navigating their medical challenges. The health care provider can facilitate an active role for the patient in their continuing wellness, including directing them to the use of specialists and reliable online resources to help them learn more. Engaging the patients to positively approach their medical challenges through education will empower the patients.

Trained medical professionals can use readily available screening tools. Only 10 minutes or less is required for the screening to assess the individual for cognitive impairment. Though we know that screening tools alone are insufficient and do not provide a diagnosis of Alzheimer's disease, they are the most critical first step to objectively determine if a cognitive change is present. Below are several possible tools to use (shortlist):

- MMSE–Mini Mental State Examination (MMSE)
- Mini-Cog–Mini Cognition
- MIS–Memory Impairment Screen
- MOCA–Montreal Cognitive Assessment
- SLUMS–the Saint Louis University Mental Status Examination
- GPCOG–General Practitioner assessment of Cognition
- AD8–AD8 Informant Interview
- The Clock Drawing Test for Alzheimer's
- Short IQCODE–Short Form of the Questionnaire on Cognitive Decline in the Elderly
- RUDAS–Rowland Universal Dementia Assessment Scale

The National Institute on Aging (NIA) does not endorse specific screening tools. The choice you make for the screening tool depends on and can range from the setting you will screen in, demographics and language, the target audience, population age and the administrator's expertise in the screening tests. Cognitive assessments can be performed on any visit. However, it is now a required component of the Medicare Annual Wellness Visit. Coverage for annual wellness visits and follow-up visits for cognitive assessment and care plan service is extended to patients who have had Medicare Part B coverage for at least 12 months. (It is recommended that you contact the Medicare and Medicaid offices to request the billing code.) The staff must be adequately trained to administer these screening tools. Therefore, it may be best to have selected individuals adequately trained and available to administer the tests each and every time.

#### 3.13.1. Who can use these tests?

- Nurses
- Speech-Language Pathologists
- Occupational Therapists
- Nurse practitioners
- Physician assistants
- Neurologists
- Psychologists
- Geriatricians
- Physicians (Primary Care Physicians or Specialist Physicians) and more

#### 3.13.2. Most popular cognitive tests

**MMSE:** The Mini-Mental State Examination (MMSE) is a widely used and reliable tool for detecting dementia. Taking about 10 minutes to complete, the MMSE measures various aspects of cognition, including language abilities, orientation, word recall, attention and calculation, language abilities and visual construction. MMSE is managed by Psychological Assessment Resources (PAR). You are required to register, complete a four-page permissions request form and pay a fee for each MMSE form and its use (approximately $1.35). *Scores may need to be adjusted or interpreted differently to account for a person's age, educational level and ethnicity/race* [27].

**Mini-Cog:** The Mini-Cog is a rapid Alzheimer's screening test that takes approximately 3–5 minutes to administer. It combines a 3-item recall with the clock-drawing test and can assist in determining whether a person has or does not have dementia. This test is widely used, and reports have been positive. This test, like the other tests, does not provide a diagnosis. There is still the requirement to conduct a thorough diagnostic work-up [28].

**Memory Impairment Screen (MIS):** The memory impairment screen (MIS) is a brief screening tool to assess memory. It is often used as a preliminary test with other screening tools to evaluate the cognition of someone who seems to display some possible impairment in their ability to think and recall [29].

**MoCA:** The Montreal Cognitive Assessment (MoCA) is a relatively simple, brief test that helps health professionals quickly determine if a person has declining cognitive function. This test may require a more thorough diagnostic workup to determine Alzheimer's disease. Unlike the MMSE, the MoCA includes a clock-drawing test and an executive function test known as Trails B. This test has been shown to have the ability to identify cognitive problems in people and may predict dementia in people with mild cognitive impairment (MCI) [30],[31]. It has been shown to identify cognitive problems in people with Parkinson's disease and other neurodegenerative disorders. To administer the MoCA assessment requires the administrator to hold a training certificate from MoCA©. *Written permission and Licensing Agreement is required if funded by commercial entity or pharma. MoCA© *may be used, reproduced and distributed **WITHOUT** permission. The test should be made available free of charge to patients* [30],[31].

**SLUMS:** The Saint Louis University Mental Status Exam (SLUMS) is an 11-item Alzheimer's screening test that is especially good at identifying people with milder cognitive problems that don't yet rise to the level of dementia [32]. This test includes items such as naming animals (similar to verbal fluency and recognition of geometric figures).

**BAS:** This Brief Alzheimer's Screening Test (BAS) is a short screening test that asks the individual to repeat three words immediately after hearing them; they are given two tasks that would distract from those three words, followed by a short version of the verbal fluency test where the individual is asked to name as many animals as they can in 30 seconds and spelling “WORLD” backward. Finally, the individual is asked to remember and recite the three words from the beginning of the screening process [33].

**GPCOG:** The GPCOG is a reliable, valid and efficient instrument to screen for dementia. The GPCOG has demonstrated that it performs at least as well as the standard screening tool, the Mini-Mental State Examination (MMSE) [34]. Testing takes only 4 minutes to administer the patient assessment and 2 minutes to interview the caregiver using the Informant interview form. Reviews of dementia screening tools for the primary care setting recommend using the GPCOG [34]. The GPCOG score is not influenced by a person's cultural and linguistic background, making it an invaluable screening tool, especially in multicultural patient settings [34],[35].

**AD8 Informant Interview:** The AD-8 Informant interview is an 8-item questionnaire that can distinguish between people who have dementia and people who don't. It is an informant-based assessment, as it is the patient's informant (usually a spouse, child or non-family caregiver) who is asked to assess whether there have been changes in some regions of cognition and functioning in the past few years [36]. These include memory, orientation, executive function and interest in activities. The AD8 has a yes or no format and takes only 3 minutes to complete.

**The Clock-Drawing Test for Alzheimer's:** The Clock Drawing Test for Alzheimer's is a simple test used with other Alzheimer's screening tests. The individual is asked to draw a clock, write all the numbers and set the hands “to a certain time”. Clock drawing tests suggest problems with memory, executive function or visuospatial abilities [37].

**Short IQCODE:** The Informant Questionnaire on Cognitive Decline in the Elderly (IQCODE) is a screening tool. It is a short questionnaire designed to assess cognitive decline and dementia in older adults. The questionnaire is filled out by a relative or friend who has known the older adult for ten years [38].

**Rowland Universal Dementia Assessment Scale (RUDAS):** The Rowland Universal Dementia Assessment Scale (RUDAS) is a short cognitive screening instrument designed to minimize the effects of cultural learning and language diversity on assessing baseline cognitive functioning. This is a multicultural cognitive assessment scale. When administering the RUDAS, the respondent must be encouraged to communicate in the language with which they are most competent and comfortable [39].

### 3.14. Barriers to cognitive testing

#### 3.14.1. Stigma

Alzheimer's disease-related stigma is often associated with loss of identity and independence and incorrect assumptions about an individual's mental and physical functioning from the moment the individual has been diagnosed [40]. Stigma associated with Alzheimer's and related dementia creates a barrier to early detection, diagnosis, care and support. The stigma of Alzheimer's is also prevalent in the health care system, particularly among health care professionals, including physicians. Often, physicians are reluctant to diagnose Alzheimer's or dementia due to not knowing much about the disease, fear of giving a death sentence to the older adult, stigma, and judgment of the illness, not understanding how various cultures view aging or mental health and not knowing what the next step of the assessment should be after the testing.

#### 3.14.2. Culture really matters

Health inequities cause significant challenges in the medical community, but health care professionals, including physicians, hold a powerful tool at their disposal: *Cultural Competence*. Social determinants of health, such as a person's living and working conditions and the quality of access to health care, aren't the same for everyone. Cultural competence in the medical field addresses the disparities that people of racially and culturally diverse backgrounds often experience. It is crucial to ensure that all individuals receive the care needed to live healthier lives. Cultural competence in health care services may improve communication and collaboration between the physician and patient, increase and improve patient satisfaction and enhance adherence, thereby improving medical outcomes and reducing health disparities [40]. This information on cultural competency for physicians aims to promote an active and united approach to multicultural health issues in the medical field and medical school training. Interventions focused on cultural competence can positively impact health professionals' knowledge, attitudes and skills, including patient satisfaction, understanding of the disease and increased likelihood of participating in preventative care and lifestyle modifications [40],[41]. Cultural competence in health care means delivering effective, quality care to patients with diverse beliefs, attitudes, values and behaviors. This also requires an understanding of any potential impact that cultural differences can have on health care delivery.

#### 3.14.3. What is cultural competence in health care?

Cultural competence in health care delivers practical and quality care to individuals of diverse backgrounds, beliefs, values and behaviors. This includes people from distinctive ethnic and racial groups, those from diverse socioeconomic backgrounds, people with disabilities and members of the LGBTQ+ communities [40],[41]. The medical field must offer personalized care and support to these diverse individuals according to their cultural and linguistic differences. It also requires that physicians and other health care professionals understand the potential impact that cultural differences can have on their health care delivery to those different than themselves. For example, an individual's race, ethnicity, socio-cultural beliefs and traditions, socioeconomic status, health literacy, and some other factors can influence their care, including

- The individual's perception of their symptoms and how it affects their health
- How and when the individual seeks care
- The individual's expectation of care they seek
- The individual's choice and preference for care treatments
- The individual's readiness to follow the physician's recommendations or treatment plans
- Who the individual believes should be involved with making medical decisions for them

#### 3.14.4. Improving cultural competence in health care

For the physician and their medical team to meet the needs of all patients, they must learn how to improve cultural competence in health care. They can then begin implementing strategies to develop and enhance cultural competence among their health care teams. Here are a few strategies to begin the process of removing the barriers to increasing cognitive assessments within diverse communities:

- Promote awareness and education on the importance of cultural competency
- Medical professionals need to identify their own beliefs and build an awareness of their own cultural biases and microaggressions
- Be open to unfamiliar attitudes, practices, beliefs, biases, microaggressions and behaviors on both sides of the table (It can improve collaboration with patients and help them to respond with flexibility)

#### 3.14.5. Benefits of cross-cultural awareness

- Improved rapport
- Person-centered recommendations and treatment plans
- Improved patient participation and compliance

### 3.15. Removing the barriers to screening

#### 3.15.1. Communicating with an older adult

Good communication is an integral part of the healing process. Many patients may prefer direct responses to the changes they are experiencing, while some prefer a reserved and cautious approach [42]. With effective communication, physicians or other health care professionals can dramatically improve their skills of speaking with, assessing and establishing more satisfying relationships with their patients. Effective communication can also help prevent medical errors, improve health outcomes, make the most out of restricted interaction time and strengthen patient and caregiver relationships [43],[44]. Here are a few tips for establishing your relationship with an older adult:

- Establish respect immediately by using formal language (Such as using Mr., Mrs., Ms., Dr.), and then let them guide you about how they would like to be addressed after that or ask your patient how they would like to be addressed.
- Be candid when speaking to an older adult about their cognitive decline or Alzheimer's disease. Truth-telling is an essential component but being respectful of the person's dignity and emotions is equally crucial. While it is necessary to be honest, avoid conflict that could lead to resistance. With resistance, you will get nowhere.
- Introduce yourself clearly but not too quickly to both the patient and caregiver.
- Speak slowly instead of speaking at a rapid-fire pace, as older adults may have difficulty following.
- They need time to process information that is being asked or said.
- It is okay to ask friendly questions about their families and activities, as this can relieve stress. Often this only takes 20–30 seconds, but it can go a long way in helping build rapport.
- Ensure that your waiting area has comfortable seats for the older adults.
- Inform staff of the importance of being mindful of the older adult patients who may need to be escorted to and from the offices, restroom, exam room and waiting area.
- Staff should check on older adults often, especially if they have a long wait in the exam room.
- Build rapport with both the patient and caregiver.
- Show that you are interested in the patient and their concerns from the start. Even if the caregiver is there, direct your attention and questions to the patient first and then to the caregiver (i.e., address the patient directly, even if the caregiver is answering the questions). This engages the patient and makes it more likely that they answer the question in their own words.

#### 3.15.2. Finding the time to screen

Make cognitive screening a part of your standard clinical protocol:

- Provide standard education on cognitive changes as people age.
- Become comfortable asking your patients about cognitive symptoms and ask every patient at some point in the year.
- Screen for memory loss and change in thinking patterns for older adults by incorporating screening tools or questionnaires.
- It is essential to screen because of inadequate detection due to a mismatch in the expectations between patients and their physicians.
- Cognitive screenings should be a routine part of any primary care visit for older adults aged 65 and older.
- Remember that culture does matter.

#### 3.15.3. Disclosing the diagnosis

In a clinical practice setting, disclosure refers to the diagnosis of dementia and an explanation of the specific disease identified through the diagnostic evaluation as the most likely cause of dementia.

- The diagnosis of Alzheimer's disease or related dementia is when the disorder most commonly causing dementia syndrome is disclosed to patients and caregivers.
- Diagnosis is disclosed to patients and caregivers less than 50% of the time.
- Withholding the diagnosis of dementia and its specific disease is a significant disadvantage to many patients and their families. Non-disclosure delays their access to available services and treatments to improve their cognition, assist them in planning for their future and enhance their quality of life.
- Be honest and informative but watch for the patient's and family's responses. They may not be ready to hear all the information at once.

### 3.16. Medication list review

The field of medicine is not fixed. The medical field experiences constant changes, additions, reviews and recalls. A patient's conditions also change. Medication review is a necessary task in helping to prevent adverse reactions from medications, prevent adverse drug combination interactions, improve the patient's medical condition, remove ineffective medications and reduce the usage of medicines to save money. Reviewing and discussing medications with your patient may help you to

- Respond to the patient's questions
- Verify their medications
- Identify medication interactions and medication errors

#### 3.16.1. Action: Do a brown bag check

A brown bag check is critical to the care of your patient. Ask your patient to bring to their appointment all medications and over-the-counter supplements. Conducting the brown bag review can be an eye-opening experience for many physician offices. Here are a few tips to get patients to bring all medications and supplements:

What to bring: Review with patients what to bring

- All prescription medicines (including pills, drops, inhalers and creams)
- All over-the-counter medicines and supplements they take regularly
- All vitamins and supplements
- All herbal medicines

Ways to remind

- On the appointment card
- During the appointment reminder call
- During the visit: discuss as a part of their visit
- Hang posters in the exam rooms and the waiting room
- Bulletin board: Display a bulletin board with anonymous case studies and persuasive reasons for bringing in their medications
- Emphasize medication reduction: A brown bag review may result in the physician stopping some medications, which is often appealing to patients
- Provide a carrier: Consider providing your patients with a small bag (canvas, paper or plastic) to carry their medications. The bag may have a printed reminder on one side and your practice name on the other.

Set out the medications

- The nurse should place all the patient's medications on the counter in the exam room to remind the clinician to perform a medication review.

Review the medications

- Introduce the review process: Ask the patient if they have any questions about their medications and verbally acknowledge the purpose of reviewing medications.

Clarify medication instructions

- Review what medications they should be taking now and how to handle them with the patient. Use the “teach-back method” to confirm understanding.

### 3.17. Terminology

**Alzheimer's disease (AD).** A progressive degenerative brain disease that causes memory loss, impaired thinking, impaired judgment, personality changes and global loss of cognitive abilities. It is the most common dementia. The primary risk factor for this condition is age—the primary prevalence of Alzheimer's disease is for 60 and above. Impairment is irreversible.

**Assessment.** It is the collection of symptoms from both the person and the informant. The collection of symptoms includes cognitive signs and functional symptoms, with screening for objective cognitive change through a brief test to determine if there is a potential presence of impairment. This will also include a “full workup”/complete medical examination (to include mood screening, more focused cognitive history, medication review, laboratory testing (looking for reversible causes) and brain imaging). Some of this is likely done by a specialist.

**Cognition.** The mental process of knowing, thinking, learning, remembering, being aware of surroundings and using judgment; it is the psychological result of perception, learning and reasoning.

**Cognitive screening/testing.** Cognitive testing checks for problems with cognition. Cognitive testing is often used to screen for mild cognitive impairment (MCI). Cognition is a combination of processes in your brain that involves most aspects of a person's life. It includes thinking, memory, language, judgment and the ability to learn new things. If there is a problem with cognition, it is called cognitive impairment. The condition ranges from mild to severe.

**Creutzfeldt-Jakob disease (CJD).** Creutzfeldt-Jakob disease (CJD) is a rare, degenerative brain disorder. Symptoms usually start around age 60. Memory problems, behavior changes, vision problems and poor muscle coordination progress quickly to dementia, coma and death. Most patients die within a year. CJD can either be sporadic, hereditary or acquired. Impairment is irreversible.

**Cultural competency.** Cultural and linguistic competence is a set of behaviors, attitudes and policies that come together in a system or agency or among professionals, enabling them to work effectively in cross-cultural situations. Competence implies the capacity to function effectively as an individual and an organization within the context of the cultural beliefs, behaviors and needs presented by your patients, consumers and their communities. It is the ability to understand, interact with and work well with people of different cultures. In medicine, the main goal of cultural competency is to help ensure health care quality is equal among all cultural groups.

**Dementia.** The term dementia is the umbrella term for the decline in mental functioning. There are over 400 known types of dementia. Functions affected by dementia include memory, language skills, problem-solving and the ability to concentrate, with personality changes, emotional problems, difficulties with activities of daily living (ADL), loss of judgment and affected visual and spatial perception. The most common form of dementia is Alzheimer's disease. There are reversible and irreversible dementias. Irreversible dementia usually worsens over time.

**Diagnosis of dementia.** Dementia is diagnosed by clinical assessment, a process to identify the disease or condition explaining the symptoms occurring in a patient. The information required for diagnosis is collected from a historical and physical examination of the patient and preferably confirmed by one or more diagnostic procedures such as cognitive and neurological tests, brain scans, psychiatric evaluation, genetic tests and blood tests.

**Frontotemporal dementia (FTD).** A syndrome caused by progressive degeneration of the frontal or temporal lobes of the brain. Frontotemporal dementia (FTD) is often referred to as Pick's disease and is a sporadic and hereditary disorder that affects the frontal and temporal lobes. FTDs include behavior variant FTD, primary progressive aphasia and FTD with motor neuron disease. It is manifested with personality changes and deterioration of language skills. This rare form of dementia tends to occur in people younger than 60. Impairment is irreversible. Pick's disease is a type of frontotemporal dementia where one's brain cells gradually stop working, and those areas shrink.

**Geriatric assessment.** An evaluation of physical, physiological or mental functioning in the older adult population group. It is the complete examination of an older adult, which includes a total evaluation of their physical and cognitive conditions and a check of the patient's emotional state.

**HIV-associated dementia.** This rare disease is when the HIV virus spreads to the brain. Impairment is irreversible.

**Huntington's disease (HD).** An inherited, progressive brain disease. Impairment is irreversible.

**Lewy body dementia (DLB).** This progressive form of dementia is characterized by abnormal amounts of protein deposits (alpha-synuclein) in the brain. These deposits of protein are called Lewy bodies. It occurs in the midbrain and cerebral cortex and causes loss of cholinergic and dopaminergic neurons. The signs and symptoms overlap with Alzheimer's and Parkinson's disease. Impairment is irreversible.

**Mild cognitive impairment (MCI).** MCI is when a person may experience more memory loss or thinking problems than the average person their age. The symptoms of MCI are not as severe as those of Alzheimer's disease or related dementia. Individuals with MCI are usually able to care for themselves and carry out their activities of daily living.

**Mixed dementia.** Mixed dementia is a condition in which a person has more than one type of dementia. Alzheimer's disease and vascular dementia is the most common type. Impairment is irreversible.

**Parkinson's disease.** It is a progressive degenerative disorder of the central nervous system. Due to nerve cell damage in the brain, dopamine levels drop, resulting in the symptoms of Parkinson's. Parkinson's disease is frequently accompanied by MCI and dementia, as the disease is progressive. Executive dysfunction will also develop. Cognitive changes experienced with Parkinson's are difficulty planning and accomplishing tasks and feelings of distraction and disorganization. Visual-spatial function may be impaired, along with short-term memory. Parkinson's disease can also affect one's movement. Signs and symptoms include tremors which are often most noticeable during rest, muscle rigidity, slowing of voluntary movements, a tendency to fall back and a mask-like facial expression. Impairment is irreversible.

**Reversible dementias.** These conditions may be associated with cognitive or behavioral symptoms that can be resolved once the primary cause is treated.

**Vascular dementia.** Vascular dementia is the second most common dementia in older adults. It is a degenerative condition. This dementia is used to describe problems with planning, judgment, reasoning, memory and thought processes caused by conditions that cause brain damage in the blood vessels in the brain or interrupt the flow of blood and oxygen to the brain. It leads to reduced brain activity. Impairment is irreversible.

## 4. Discussion

Efforts in the United States have been limited in scope when it comes to improving cognitive impairment care related to dementia in older adult Americans. There are many barriers within the healthcare systems and amongst the health care professionals that have yet to be addressed. The creation of a national strategy with mandated education for health care professionals will provide clinicians and other medical professionals with sustainable improvements, including knowledge and transferable skills for dementia detection, diagnosis and care. Best practice should include and embrace early detection and diagnosis. Currently, diagnosis occurs during the time of a family crisis.

Removing the barriers that could lead to social change in the medical field's response to early detection, diagnosis, disclosure and care will require mandated dementia education that is specific, detailed, consistent and substantial. We need to improve further education that surpasses the formal education that only touches on the pharmacology and pathophysiology in formal dementia education. Educational interventions require a broader scope that addresses the gaps related to clinician attitudes, knowledge, skills, and behaviors.

It is important for the creation and dissemination of new knowledge using revised or modernized cognitive assessment tools. These assessment tools should consider the many factors in aging, such as age, education, culture, race, sexual identity, language, attitude, beliefs about the symptoms of dementia, vision and hearing impairment and illiteracy or lower education levels. At this time, it is now critical to have effective and efficient transferal of gained knowledge to clinician practices and interventions that are tangible, substantial, and sustainable. Without the acknowledgement and action based on clinical recommendations on dementia education, Alzheimer's disease and related dementias will become more of an uncontainable problem, with increased under-detection, misdiagnosis and untreated illnesses contributing to the escalation of other health and financial issues.

## 5. Implications for advanced clinician knowledge and practice

This article addressed the current and ever-growing challenges faced by primary care physicians and other clinicians regarding the current dementia care knowledge and attitudes that exist in the medical field. There is an urgent and critical need for removing the barriers to dementia early detection, diagnosis and care. In the article “The Knowledge and Attitudes of Primary Care and the Barriers to Early Detection and Diagnosis of Alzheimer's Disease,” the author summarized the need to improve and increase the training for physicians, medical students, and other medical staff. Training needs to be accessible to all professionals working with the older adult population. The article also discussed the need for the implementation of national or statewide dementia educational programs that enhance the skills of clinicians. Because the medical field is not static, from medical school to clinical practice, dementia education should be ongoing. This action will ensure that there is long-term sustainability in dementia care and dementia education programs.

## 6. Limitations

One of the limitations of this article is that it is in the form of an informative and actionable toolkit. It is very specific to primary care physicians and other clinicians. Though other medical staff and other health care professionals would benefit from this information, they were not included. However, they would greatly benefit from the newly generated knowledge in dementia care, as it would enhance their knowledge and care practices.

## 7. Recommendations

Cognitive impairment is a medical condition. It is imperative to acknowledge, understand and act, as cognitive impairments, whether reversible or irreversible, require the attention of clinicians and others in the health care community. The Medicare Annual Wellness Visit (AWV) was implemented to include cognitive assessments since cognitive impairments, AD and other dementias have been under-detected and under-diagnosed. The 2019 Alzheimer's Association Report states that approximately only 50 percent of patients use their primary care physician to assess their cognitive functioning. The intention of this article is to broaden the knowledge of the barriers to early detection, diagnosis and care within the medical field so that primary care physicians and other clinicians can use it to understand and improve their cognitive screening knowledge, accuracy and confidence and advocate for more valuable and equitable dementia care education to care for the diverse population of older adults. Education of removing the barriers to detection and diagnosis of cognitive impairments and understanding the diseases, causes and stages in older adults should be requirements for medical professionals. Cognitive assessments should be a standard protocol for primary care professionals caring for older adults.

## 8. Conclusions

This toolkit can act as an educational intervention in primary care practices to improve the rates of, accuracy of and access to cognitive assessments for brain health decline. Reducing the barriers to early detection and diagnosis will lead to accurate care and reduce the gaps in detection. This toolkit is an introduction to reducing the prevalent gaps, and more work is required to overcome barriers to the development and implementation of educational interventions that increase feasibility in medical practices.

## References

[1] Code of Federal Regulations (2022). Title 42 CFR 410.15(a) Detection of any cognitive impairment.

[2] Alzheimer's Foundation of America (AFA) (2022). What is a memory screening?.

[3] Centers for Disease Control and Prevention (CDC) (2022). What is dementia.

[4] Jessen F, Amariglio R, Buckley R (2020). The characterization of subjective cognitive decline. Lancet Neurol.

[5] Cullen NC, Leuzy A, Palmqvist S (2021). Individualized prognosis of cognitive decline and dementia in mild cognitive impairment based on plasma biomarker combinations. Nat Aging.

[6] Jessen F, Amariglio RE, van Boxtel M (2014). A conceptual framework for research on subjective cognitive decline in preclinical Alzheimer's disease. Alzheimers Dement.

[7] Chen YX, Liang N, Li XL (2021). Diagnosis and treatment for mild cognitive impairment: A systematic review of clinical practice guidelines and consensus statements. Front Neurol.

[8] National Institute of Aging (2022). Alzheimer's Disease Fact Sheet.

[9] Livingston G, Sommerlad A, Orgeta V (2017). Dementia prevention, intervention, and care. Lancet.

[10] Alzheimer's Association (2022). 2022 Alzheimer's disease facts and figures. Alzheimers Dement.

[11] Dafsari FS, Jessen F (2020). Depression—an underrecognized target for prevention of dementia in Alzheimer's disease. Transl Psychiatry.

[12] Aminzadeh F, Molnar FJ, Dalziel WB (2012). A review of barriers and enablers to diagnosis and management of persons with dementia in primary care. Can Geriatr J.

[13] Chai DSC (2013). Early diagnosis of dementia in the primary care setting. Singapore Fam Physician.

[14] Staffaroni AM, Tsoy E, Taylor J (2020). Digital Cognitive Assessments for Dementia: Digital assessments may enhance the efficiency of evaluations in neurology and other clinics. Pract Neurol.

[15] Tsoy E, Sideman AB, Piña Escudero SD (2021). Global perspectives on brief cognitive assessments for dementia diagnosis. J Alzheimers Dis.

[16] Judge D, Roberts J, Khandker R (2019). Physician perceptions about the barriers to prompt diagnosis of mild cognitive impairment and Alzheimer's disease. Int J Alzheimers Dis.

[17] Dubois B, Padovani A, Scheltens P (2016). Timely diagnosis for Alzheimer's disease: A literature review on benefits and challenges. J Alzheimers Dis.

[18] de Levante Raphael D (2022). The knowledge and attitudes of primary care and the barriers to early detection and diagnosis of Alzheimer's disease. Medicina.

[19] Mansfield E, Noble N, Sanson-Fisher R (2019). Primary care physicians' perceived barriers to optimal dementia care: a systematic review. Gerontologist.

[20] Collins R, Hunt A, Quinn C (2022). Methods and approaches for enhancing communication with people with moderate-to-severe dementia that can facilitate their inclusion in research and service evaluation: Findings from the IDEAL programme. Dementia.

[21] Dooley J, Bailey C, McCabe R (2015). Communication in healthcare interactions in dementia: a systematic review of observational studies. Int Psychogeriatr.

[22] American Family Physician (2020). Screening for Cognitive Impairment in older adults: Recommendation statement. Am Fam Physician.

[23] de Levante Raphael D (2022). Stigmas of Alzheimer's disease and help seeking for Alzheimer's disease among African Americans. Walden University ProQuest Dissertation Publishing.

[24] Yiannopoulou KG, Papageorgiou SG (2020). Current and Future Treatments in Alzheimer Disease: An Update. J Cent Nerv Syst Dis.

[25] Atri A, Shaughnessy L, Locascio J (2008). Long term course and effectiveness of combination therapy in Alzheimer's disease. Alzheimer Dis Assoc Discord.

[26] Porsteinsson AP, Isaacson RS, Sabbagh MN (2021). Diagnosis of early Alzheimer's disease: Clinical practice in 2021. J Prev Alzheim.

[27] Myrberg K, Hydén LC, Samuelsson C (2019). The mini-mental state examination (MMSE) from a language perspective: an analysis of test interaction. Clin Linguist Phonet.

[28] Mini-Cog Quick screening for early dementia detection.

[29] Buschke H, Kuslansky G, Katz M (1999). Screening for dementia with the memory impairment screen. Neurology.

[30] Montreal Cognitive Assessment (MoCA) (2022). https://mocatest.org.

[31] Hoops S, Nazem S, Siderowf AD (2009). Validity of MoCA and MMSE in the detection of MCI and dementia in Parkinson disease. Neurology.

[32] SLU Mental Status Exam (2022). https://www.slu.edu/medicine/internal-medicine/geriatric-medicine/aging-successfully/assessment-tools/mental-status-exam.php.

[33] Mendiondo M, Ashford JW, Schmitt F (2003). Designing a Brief Alzheimer Screen (BAS). J Alzheimers Dis.

[34] General Practitioner Assessment of Cognition (2022). http://gpcog.com.au/.

[35] Yokomizo JE, Seeher K, de Oliveira GM (2018). Cognitive screening test in primary care: Cut points for low education. Rev Saude Publica.

[36] Galvin J, Zweig Y (2021). The AD8: The Washington University Dementia Screening Test.

[37] Kim S, Jahng S, Yu KH (2018). Usefulness of the clock drawing test as a cognitive screening instrument for mild cognitive impairment and mild dementia: an evaluation using three scoring systems. Dement Neurocogn Disord.

[38] Ding Y, Niu J, Zhang Y (2018). Informant questionnaire on cognitive decline in the elderly (IQCODE) for assessing the severity of dementia in patients with Alzheimer's disease. BMC Geriatrics.

[39] Dementia Australia, Rowland Universal Dementia Assessment Scale (RUDAS).

[40] Kumar R, Bhattacharya S, Sharma N (2019). Cultural competence in family practice and Primary care setting. J Family Med Prim Care.

[41] Stubble D (2020). Practicing cultural competence and cultural humility in the care of diverse patients. Focus.

[42] Connel CM, Boise L, Stuckey JC (2004). Attitudes toward the diagnosis and disclosure of dementia among family caregivers and primary care physicians. Gerontologist.

[43] Huang SC, Morgan A, Peck V (2021). Improving communications with patients and families in geriatric care. The how, when and what. J Patient Experience.

[44] Alzheimer's Association (2019). New Alzheimer's Association report shows significant disconnect between seniors, physicians when it comes to cognitive assessment.

